# Physical Activity and Sedentary Behavior Associated with Components of Metabolic Syndrome among People in Rural China

**DOI:** 10.1371/journal.pone.0147062

**Published:** 2016-01-20

**Authors:** Jing Xiao, Chong Shen, Min J. Chu, Yue X. Gao, Guang F. Xu, Jian P. Huang, Qiong Q. Xu, Hui Cai

**Affiliations:** 1 Department of Epidemiology and Medical Statistics, School of Public Health, Nantong University, Nantong, Jiangsu, 226019, China; 2 Department of Epidemiology, School of Public Health, Nanjing Medical University, Nanjing, Jiangsu, 210029, China; 3 Department of Chronic Disease and Prevention, Center for Disease Control and Prevention of Nantong, Nantong, Jiangsu, 226007, China; Universidad Pablo de Olavide, Centro Andaluz de Biología del Desarrollo-CSIC, SPAIN

## Abstract

**Background:**

Metabolic syndrome is prevalent worldwide and its prevalence is related to physical activity, race, and lifestyle. Little data is available for people living in rural areas of China. In this study we examined associations of physical activity and sedentary behaviors with metabolic syndrome components among people in rural China.

**Methods:**

The Nantong Metabolic Syndrome Study recruited 13,505 female and 6,997 male participants between 2007 and 2008. Data of socio-demographic characteristics and lifestyle were collected. The associations of physical activity and sedentary behaviors with metabolic syndrome components were analyzed.

**Results:**

Prevalence of metabolic syndrome was 21.6%. It was significantly lower in men than in women. Low risks of metabolic syndrome were observed in those who did less sitting and engaged in more vigorous physical activity. The highest tertile of vigorous physical activity was associated with 15–40% decreased odds of metabolic syndrome and all of its components, except for low high-density lipoprotein cholesterol in men. Women with the highest tertile of moderate physical activity had 15–30% lower odds of central obesity, high glucose, and high triglycerides compared with those in the lowest tertile. Sitting time >42 hours per week had a 4%-12% attributable risk of metabolic syndrome, central obesity, and high triglycerides in both genders, and abnormal glucose and diastolic blood pressure in women. Sleeping for more than 8 hours per day was associated with risk of high serum glucose and lipids.

**Conclusions:**

Our data suggested that physical activity has a preventive effect against metabolic syndrome and all its abnormal components, and that longer sitting time and sleep duration are associated with an increased risk of metabolic syndrome components, including central obesity and high triglycerides, glucose, and diastolic blood pressure. This study could provide information for future investigation into these associations. Also, recommendations are developed to reduce prevalence of metabolic syndrome and its components in rural Chinese populations.

## Introduction

Metabolic syndrome (MS) is a cardio-metabolic risk cluster comprising high fasting glucose level, hypertension, high triglyceride level, low high-density lipoprotein cholesterol (HDL-c) level, and high waist circumference [1], and is strongly associated with the development of type II diabetes and risk of cardiovascular morbidity and mortality [2]. MS is highly prevalent in adult populations worldwide, with data suggesting an ethnic predisposition in Asian populations [3]. It is also closely associated with lifestyles, such as smoking, drinking, physical activity (PA), and sedentary behavior.

The World Health Organization estimates that physical inactivity causes approximately 22% of ischemic heart disease, 10–16% of diabetes, and 2 million deaths worldwide, annually [4]. Many epidemiological studies have reported a significantly negative association between the levels of PA and the incidence of MS in both adults [5] and children [6], especially in the association of moderate to vigorous PA with MS and its components [7–10]. However, the data from these studies are inconsistent. A meta-analysis of cohort studies reported that moderate exercise at leisure time was weakly associated with a decreased risk of MS in men [11]. Other studies found that this exercise had a protective effect on hypertension in Brazilian men [12], little or no effect on Korean adults [13]. It has also been reported that total PA was associated with a lower prevalence of MS in Portuguese adults [14] but was not associated with MS in the Costa Rican population [14, 15]. These inconsistencies could also be found in the association of light PA with MS and its components [16, 17]. In our study PA refers to both leisure time physical activity and occupational physical activity. Sedentary behavior refers to any waking activity characterized by energy expenditure ≤ 1.5 metabolic equivalents and a sitting or reclining posture [18]. Sedentary behavior have been shown to be positively associated with an increased risk of MS [19], and this association between sedentary behavior and the MS is independent of levels of PA [20]. It suggests that sedentary time could be an independent determinant of metabolic dysfunction distinct to that of physical activity. But other report didn’t support this opinion [21]. Also sedentary behavior was associated with increasing body mass index (BMI) and waist circumference in US adults [22], and an increased risk of type 2 diabetes in a systematic review of prospective studies [23]. These inconsistent reports could be due to different populations or relatively small sample size in each study. Also we did not found any data of association of different intensities of PAs or sedentary behaviors with each of the MS components, especially in rural China.

We launched the Nantong Metabolic Syndrome Study in 2007. It is a large population-based cross-sectional study of 20,502 participants (including 6,997 men and 13,505 women), aged 18–74 years, in the rural areas of Nantong, China. The objective of this study was to fully assess the associations of different PA intensity and sedentary behavior with each of the MS components among people in rural China respectively because PA is a protective factor and sedentary behavior is a risk factor in link with MS in previous studies. To our knowledge, this is the largest study in rural China.

## Materials and Methods

### Study population

The Nantong Metabolic Syndrome Study recruitment began in July 2007 and was completed in August 2008. The details of it have been described elsewhere [24, 25]. Briefly, 24,519 residents between the ages of 18 and 74 years were recruited from two townships in rural Nantong. Among them, 20,502 participants (6,997 men and 13,505 women) were enrolled in the study, with a response rate of 83.6%. The reasons for non-participation were refusal (3.21%), being out of the area during enrollment (7.21%), and other miscellaneous reasons such as poor health or hearing problems (5.98%). We defined a rural area as an area with a primary administrative unit termed a ‘village’. At the time of interview, most participants (99.5%) lived in a village, and 13,306 (64.9%) were farmers, most of whom were vigorous physical laborers. The Boards of Scientific Research of Nantong University and the Nantong Centers for Disease Control ethics review board approved the original survey protocols and all participants provided their written consent to participate in this study. We assessed the socio-economic factors, dietary intake, alcohol consumption, smoking status, physical activity, sedentary behavior, personal medical history, and family history of several chronic diseases, using the same standard questionnaires for all participants.

### Physical activity and sedentary behavior assessment

PA level was assessed in our study using a questionnaire, which is similar to the International Physical Activity Questionnaire, a standardized questionnaire used in epidemiological studies [26]. We collected data on the cumulative hours per day for light, moderate and vigorous intensity PA in two domains (occupational PA and leisure-time PA), and for sitting time, watching TV, and sleeping during the previous 7 days. Intensity Levels of leisure-time PA were defined below: no leisure-time PA (watching TV, reading and writing), light leisure-time PA (Qi Gong and some stretching exercises), moderate leisure-time PA (jogging and dancing) and vigorous leisure-time PA (playing basketball, badminton). Occupational PA was divided into four groups: No job or sedentary work (typists, computer operators), light occupational PA (clerk, teacher), moderate occupational PA (driver, electrician) and vigorous occupational PA (farmer, porter). Furthermore, we defined three categories for each of PA intensities (light, moderate, and vigorous) based on the tertiled of the hours per week of each PA intensity respectively, such as vigorous PA: low (≤ 14 h/w for women and ≤ 7 h/w for men), medium (≤ 42 h/w for both sexes), and high (> 42 h/w for both sexes). What’s more, total PA were calculated by sum of energy expenditure of three PA intensities and were presented as metabolic equivalents-hours per week (MET-h/w). MET-h/w were calculated using hours of each PA intensity per week multiplied by its energy requirement: light intensity = 3.3 METs, (based on walking METs), moderate intensity = 4.0 METs, and vigorous intensity = 8.0 METs. Sedentary behavior refers to activities that involve energy expenditure at the level of 1.0–1.5 metabolic equivalent units (METs) [18]. Sedentary behaviors includes lying down, sitting (reading, or using the computer and other forms of screen-based entertainment) and watching TV rather than physical inactivity. Physical inactivity was defined as not meeting any of the following criteria; (1) at least 30 min of moderate-intensity activity per day on at least 5 days per week (2) at least 20 min of vigorous-intensity activity per day on at least 3 days per week or (3) an equivalent combination by the WHO global health observatory data repository database (web link: http://apps.who.int/gho/data/node/main.A893?lang=en). Similar categorization was used for total PA in MET-h/w, sitting hours per week, hours of watching TV per week and sleep hours per day.

### Other environmental factors

Ever smokers were defined as participants who had smoked at least 100 cigarettes in their life time and they were asked how many cigarettes they consumed per day. All participants were asked about monthly alcohol consumption of grape wine, rice wine, beer, and liquor within the recent year. One drink was defined as consumption of approximately 0.5 ounces of pure alcohol [27]. The intake of meat, including red meat (e.g., pork, beef, and lamb), white meat (e.g., chicken, duck, and goose), and fish were investigated. Data on the amount other foods consumed, such as vegetables, fruits, and soy foods were also collected. We asked the participants how frequently (daily, weekly, monthly, yearly, or never) they consumed these food groups over the past year, followed by a question on the amount consumed in liang (1 liang = 50 grams) per unit of time. We defined tea consumption as drinking tea more than twice per week, for at least six months continuously. Socio-demographic factors, such as age at the time of interview, education (none, elementary school, middle/high school, college, and above), personal income in Yuan/month (≤ 500, 501–1000, ≥ 1001) and occupation, were used as potential confounders in the study.

### Anthropometric and biochemical measurements

Anthropometric measurements (weight, height, and waist circumference) were measured twice for each participant and a third measurement was taken if the difference between the two measurements was larger than 1 cm for height and waist circumference or 1 kg for weight, during the in-person interviews. The average value of two closest measurements of height, weight, and waist circumference were used in this study. BMI was calculated using the formula: weight (kg) divided by height^2^ (m^2^).

Systolic blood pressure (SBP) and diastolic blood pressure (DBP) were measured three times for each participant using a standardized mercury sphygmomanometer with the cuff on the right upper arm. The average of three measurements was used for analysis. A 10-mL blood sample, following an overnight fast, was drawn into an EDTA vacutainer tube at the time of the in-person interview. Serum samples were obtained by centrifugation of the blood samples. The serum levels of glucose and the lipid profiles of the 20,502 participants were measured using an automated chemistry analyzer (Hitachi 7180, Tokyo, Japan). Reagents from the Shino-Test Corporation in Japan were used to enzymatically analysis in the Nantong Centers for Disease Control. Both the inter- and intra-assay coefficients of variation were less than 3.5% for glucose, triglyceride, and HDL-c.

### Criteria for Metabolic syndrome diagnosis

MS criteria were proposed by the most recent Joint Interim Statement of multi-International organizations [28]. A diagnosis of MS was made if three or more of the following metabolic risk factors or cut-off points were present: (1) central obesity: waist circumference ≥85 cm for Chinese men and ≥ 80 cm for Chinese women; (2) high fasting triglyceride: ≥ 1.7 mmol/L or taking medication for abnormal lipid levels; (3) Low HDL-c: < 1.0 mmol/L for Chinese men and < 1.3 mmol/L for Chinese women or undergoing a specific treatment for abnormal HDL-c; (4) high blood pressure: SBP ≥ 130 mmHg or DBP ≥ 85 mmHg or taking hypertension medication; (5) high fasting glucose: ≥ 5.6 mmol/L or taking diabetes medication.

### Statistical analysis

Demographic, dietary, and lifestyle characteristics were presented as the mean ± standard deviation (or the median ± inter-quartile range) for continuous variables and as percentages for categorical variables. We compared these characteristics between MS and non-MS subjects using ANOVA for normally distributed variables and a Wilcoxon rank sum test for non-normally distributed variables. A chi-square test was used for categorical variables. Odds ratios (ORs) and 95% confidence intervals (CIs) were estimated using a common logistic regression to assess the association of PA intensity with MS and its components. Two dummy variables were created of each of PA intensity (for example: vigorous intensity PA in women, that is ≤ 42 h/w vs ≤ 14 h/w and > 42 h/w vs ≤ 14 h/w). Logistic regression model was used adjusted for potential confounders in the analysis. Ordinal variable (e.g. median hours per week for each category of PA) was used as continuous parameters for test the linear trend. The potentially confounding variables, including age at the time of interview, BMI, personal income, education, marital status, occupation, drinking status, smoking status, tea consumption, and intake of red meat, white meat, fish, soy food, vegetables, and fruits, were applied in the logistic regression model. Additionally light, moderate, and vigorous PA levels were mutually adjusted in the model. All p-values were based on two-tailed test and a p-value < 0.05 was considered statistically significant. All analyses were performed using SAS statistical software (version 9.4; SAS Institute, Cary, NC).

## Results

### Characteristics of study participants

Differences of selected demographic characteristics, lifestyle factors, anthropometric measurements, and food intake, between MS subjects and non-MS subjects, are presented in Table 1. According to the Joint Interim Statement criteria, the prevalence of MS was 21.6%, and it was 6.2% higher in women (23.7%) than that in men (17.5%) (p < 0.001). MS subjects were older, more likely to be tea consumers, had a higher weight and BMI, and had a higher income, than those non-MS subjects. Among men, MS subjects were more educated, less likely to be farmers, and had a higher marital rate than those non-MS subjects. While in women, MS subjects had less soy food and fruits intake and consumed less alcoholic beverages than non-MS subjects.

**Table 1:** Characteristics of the study subjects stratified by metabolic syndrome status and genders*.

	All participants		Women			Men	
		MS subjects (n = 3199)	Non-MS subjects (n = 10306)	p values	MS subjects (n = 1223)	Non-MS subjects (n = 5774)	p values
Age at interview (years, mean ± SD**)	54.2±0.1	57.4±0.2	51.9±0.1	<0.001	57.3±0.3	55.8±0.2	<0.001
Weight (kg, mean ± SD)	60.2±0.1	65.5±0.2	55.9±0.1	<0.001	74.9±0.3	62.0±0.1	<0.001
BMI (kg/m^2^, mean ± SD)	23.7±0.0	26.8±0.1	23.0±0.0	<0.001	26.8±0.1	22.5±0.0	<0.001
Red meat (g/day, mean ± SD)	30.0±0.6	26.9±1.9	28.4±1.1	0.500	35.6±1.2	33.2±0.6	0.067
White meat (g/day, mean ± SD)	183±0.2	17.0±0.5	17.0±0.3	0.913	22.5±0.8	20.5±0.4	0.024
Fish (g/day, median ± IQR^†^)	22.1±34.1	20.4±26.6	20.4±30.6	<0.001	22.1±34.1	22.1±34.1	0.265
Vegetables (g/day, median ± IQR)	225.0±187.5	225.0±187.5	225.0±225.0	0.083	300.0±187.5	267.9±187.5	0.147
Fruits (g/day, median ± IQR)	17.5±41.5	13.7±43.6	17.5±41.5	<0.001	21.0±41.3	17.5±41.5	0.047
Soy food (g/day, mean ± SD)	62.5±0.8	57.6±2.1	62.8±1.2	0.032	69.9±3.3	63.2±1.5	0.064
Education (%)							
Primary school/under	59.7	64.6	63.7		40.8	54.2	
Middle school	27.9	25.6	26.7		35.7	29.5	
High school/above	12.4	9.8	9.6	0.969	23.5	16.3	<0.001
Marital status (%)							
Yes	90.0	90.8	90.1		92.8	89.3	
No^#^	10.0	9.2	9.9	0.808	7.2	10.8	0.002
Income per person (%)							
≤500 Yuan	64.3	65.2	65.5		57.6	63.8	
501–1000 Yuan	29.1	28.5	29.2		30.4	28.7	
≥1001 Yuan	6.6	6.3	5.3	0.042	12.1	7.5	<0.001
Farmer (%)							
Yes	64.9	69.4	69.6		46.1	59.3	
No	35.1	30.7	30.4	0.413	53.9	40.7	<0.001
Ever smoker (%)							
Yes	18.8	3.6	4.0		45.8	48.1	
No	81.2	96.4	96.0	0.168	54.1	51.9	0.125
Tea consumption (%)							
Yes	15.2	12.7	9.7		36.7	21.4	
No	84.8	87.3	90.3	<0.001	63.4	78.6	<0.001
Alcohol consumption (%)							
Yes	25.6	9.2	12.0		52.6	53.9	
No	74.4	90.8	88.0	< 0.001	47.4	46.1	0.223

* Means or median, percentages, and their p values were adjusted for age at interview.

* * SD: standard deviation.

^†^ IQR: inter-quartile range (25-75th percentiles).

^#^ Including widowed, divorced/separated, and single.

### Association of physical activity and sedentary behavior with metabolic syndrome and its components

Table 2 shows the associations of MS with different PA intensity and sedentary behaviors. Hours of vigorous PA per week were associated with a decreased risk of MS in both men and women (ORs (95% CIs): 0.72 (0.64–0.81) for men and 0.84 (0.77–0.90) for women). An association between hours of moderate PA and MS was observed only in women, with an OR of 0.90 (95% CI: 0.85–0.95). We obtained similar results if we used MET-h/w in the analysis. Increasing MET-h/w of the total PA for both genders was associated with a decreased MS risk (ORs = 0.77 for men and 0.86 for women, both p for trend < 0.001). There was no association between hours of light PA and MS prevalence. Sitting time was associated with an increased prevalence of MS in both genders (ORs = 1.16 for both men and women, both p for trend < 0.001). Sitting time over 42h per week was associated with an increased risk of MS with its attributable risk of 11.38% for men and 10.57% for women, compared with sitting time not more than 7h per week (S1 and S2 Tables). There were interaction effects between sexes and moderate PA, sitting time and sleep duration (all p<0.05).

**Table 2:** Associations of MS with PA and other activities: MS subjects vs. non-MS subjects among rural men and women.

		Total				Women				Men		Interaction p
	MS subjects	OR (95% CI)^*b*^	p		MS subjects	OR (95% CI)^*a*^	p		MS subjects	OR (95% CI)^*a*^	p	
Vigorous PA (h/w)												
≤14	1952	1.0		≤14	1253	1.0		≤7	570	1.0		
≤42	1599	0.79(0.72–0.88)	<0.001	≤42	1301	0.84(0.74–0.95)	0.004	≤42	427	0.68(0.56–0.82)	<0.001	0.054
>42	867	0.65(0.58–0.74)	<0.001	>42	642	0.70(0.60–0.81)	<0.001	>42	225	0.53(0.42–0.66)	<0.001	
trend		0.81(0.76–0.86)	<0.001	trend		0.84(0.77–0.90)	<0.001	trend		0.72(0.64–0.81)	<0.001	
Moderate PA (h/w)												
0	2208	1.0		0	1611	1.0		0	597	1.0		
≤14	951	0.96(0.87–1.06)	0.397	≤14	711	0.96(0.85–1.08)	0.508	≤20	248	0.93(0.77–1.13)	0.474	0.006
>14	1260	0.82(0.74–0.90)	<0.001	>14	874	0.80(0.71–0.90)	<0.001	>20	378	0.84(0.70–1.00)	0.053	
trend		0.91(0.86–0.95)	<0.001	trend		0.90(0.85–0.95)	<0.001	trend		0.92(0.84–1.00)	0.054	
Light PA (h/w)												
≤14	2028	1.0		≤14	1450	1.0		≤14	578	1.0		
≤28	1269	1.02(0.93–1.12)	0.748	≤28	986	1.01(0.90–1.12)	0.896	≤27	156	1.12(0.89–1.41)	0.338	0.584
>28	1121	0.95(0.85–1.06)	0.385	>28	760	0.93(0.81–1.06)	0.275	>27	488	0.95(0.80–1.13)	0.571	
trend		0.98(0.93–1.04)	0.479	trend		0.97(0.91–1.03)	0.345	trend		0.98(0.90–1.07)	0.655	
Total PA (MET-h/w)												
≤216.2	1711	1.0		≤216.2	1108	1.0		≤212.2	546	1.0		
≤335.5	1414	0.85(0.77–0.93)	0.001	≤334.9	1091	0.89(0.79–0.99)	0.050	≤342.7	383	0.75(0.63–0.90)	0.002	0.053
>335.5	1294	0.70(0.64–0.78)	<0.001	>334.9	997	0.75(0.66–0.84)	<0.001	>342.7	294	0.59(0.48–0.72)	<0.001	
trend		0.84(0.80–0.88)	<0.001	trend		0.86(0.81–0.92)	<0.001	trend		0.77(0.69–0.84)	<0.001	
Sitting time (h/w)												
≤21	1373	1.0		≤21	1013	1.0		≤21	360	1.0		
≤42	1812	1.12(1.03–1.23)	0.011	≤42	1285	1.07(0.96–1.19)	0.206	≤42	527	1.21(1.02–1.44)	0.027	0.019
>42	1232	1.37(1.23–1.52)	<0.001	>42	897	1.35(1.20–1.53)	<0.001	>42	335	1.34(1.10–1.64)	0.004	
trend		1.17(1.11–1.23)	<0.001	trend		1.16(1.09–1.23)	<0.001	trend		1.16(1.05–1.28)	0.003	
Watching TV (h/w)												
≤7	1530	1.0		≤7	1203	1.0		≤7	327	1.0		
≤14	2048	0.96(0.88–1.05)	0.328	≤14	1489	0.98(0.89–1.08)	0.628	≤14	559	0.97(0.82–1.16)	0.735	0.056
>14	840	1.08(0.97–1.22)	0.171	>14	504	1.05(0.92–1.22)	0.462	>14	336	1.17(0.96–1.44)	0.124	
trend		1.03(0.97–1.09)	0.361	trend		1.02(0.95–2.09)	0.652	trend		1.08(0.97–1.20)	0.143	
Sleep duration (h/d)												
≤7	847	1.0		≤7	555	1.0		≤7	292	1.0		
≤8	2667	1.04(0.94–1.15)	0.465	≤8	1963	1.07(0.94–1.21)	0.309	≤8	704	0.97(0.81–1.16)	0.721	0.001
>8	905	1.16(1.03–1.32)	0.017	>8	678	1.16(1.00–1.35)	0.053	>8	227	1.20(0.95–1.52)	0.121	
trend		1.08(1.01–1.15)	0.018	trend		1.08(1.00–1.16)	0.050	trend		1.08(0.96–1.22)	0.185	

MS: metabolic syndrome; PA: physical activity; MET: metabolic equivalent.

^*a*^: adjusted for age at interview, BMI, education, marital status, personal income, occupation, smoking status, drinking status, tea consumption, and intake of red meat, white meat, fish, vegetables, fruits, and soy food, and mutually adjusted for light to vigorous PA categories

^*b*^: adjusted for the potential confounders in model *a* and sex.

h/w: hours per week; h/d: hours per day; MET-h/w: MET hours per week.

Table 3 and Table 4 show association of PA intensity and sedentary behavior with the MS components by gender. In general, increased time spent in moderate and vigorous PA per week and increased MET-h/w of total PA were both protective factors for the MS components, while increased sitting time was a risk factor for MS components in both genders. Increasing the MET-h/w of total PA and the hours of vigorous PA per week were significantly associated with a decreased risk for all MS components in both genders with ORs from 0.77 to 0.93 (all *p* for trend <0.05), with the exception of low HDL-c in men. And their attributable ‘risks’ (protective effect) of abnormal MS components were 27.60%–11.38% in men and 20.41%–7.04% in women who engaged in total PA over 334.9 MET-h/w, compared with those who engaged in vigorous PA less than 212.2 MET-h/w in men and 216.2 MET-h/w in women (S1 and S2 Tables). Furthermore, we found that over 42 h/w of vigorous PA was associated with a decreased risk of having high waist circumference (OR = 0.59), high triglyceride (OR = 0.63), high glucose (OR = 0.69), high SBP (OR = 0.69), and high DBP (OR = 0.59), compared with those who engaged in not more than 7 h/w vigorous PA in men. Similarly, we found women who engaged in more than 42 h/w of vigorous PA had a decreased risk of having high waist circumference (OR = 0.83), high triglyceride (OR = 0.79), low HDL-c (OR = 0.73), high glucose (OR = 0.59), high SBP (OR = 0.79), and high DBP (OR = 0.70), compared with women who had not more than 14 h/w vigorous PA. Meanwhile, hours of moderate PA was associated with a reduced risk of high waist circumference (ORs = 0.89 for men and 0.88 for women), high triglyceride only in women (OR = 0.91) and high glucose (ORs = 0.84 for men and 0.86 for women). Additionally, light PA was only weakly associated with a decreasing SBP in both genders (ORs = 0.90 for men and 0.94 for women). Increasing the hours of sitting, watching TV per week and sleeping per day were associated with an increased risk of several MS components in both sexes (*p* for trend <0.05). Sitting time over 42 h/w had 9.19% and 10.71% attributable risks of high waist circumference and 8.06% and 5.75% attributable risks of high triglyceride among men and women, respectively. It also had 11.61% and 7.74% attributable risks of high glucose and high DBP in women, compare with those who had sitting time below 21 h/w (S1 and S2 Tables). Increased time spent watching TV per week was associated with a risk of high waist circumference (OR = 1.20) in men and high triglyceride in women (OR = 1.09). Sleeping 8 h/d or more had 16.83% and 16.69% attributable risks of high glucose, in men and women, respectively, a 20.13% attributable risk of low HDL-c in men, and a 12.02% attributable risk of high triglyceride in women, compare with those who slept less than 7 h/d (S1 and S2 Tables).

**Table 3:** Associations of MS components with PA and other activities: abnormal MS components vs. normal MS components in men.

	Waist Circumference (cm)			Triglyceride (mmol/L)			HDL-c (mmol/L)			Glucose (mmol/L)			SBP (mmHg)			DBP (mmHg)		
	Cases	OR (95%CI)	p	Cases	OR (95%CI)	p	Cases	OR (95%CI)	p	Cases	OR (95%CI)	p	Cases	OR (95%CI)	p	Cases	OR (95%CI)	p
Vigorous PA (h/w)																		
≤7	1174	1.0		725	1.0		150	1.0		202	1.0		839	1.0		551	1.0	
≤42	1003	0.69(0.56-0.85)	0.001	510	0.76(0.64-0.90)	0.002	112	0.87(0.64-1.18)	0.371	154	0.73(0.56-0.96)	0.024	935	0.80(0.69-0.93)	0.004	489	0.77(0.65-0.92)	0.004
>42	627	0.59(0.46-0.76)	<0.001	313	0.63(0.51-0.77)	<0.001	68	0.76(0.52-1.11)	0.160	91	0.69(0.49-0.95)	0.025	560	0.69(0.58-0.82)	<0.001	270	0.59(0.48-0.73)	<0.001
trend		0.77(0.68-0.87)	<0.001		0.79(0.71-0.87)	<0.001		0.88(0.73-1.05)	0.160		0.82(0.70-0.97)	0.020		0.83(0.76-0.91)	<0.001		0.77(0.69-0.85)	<0.001
Moderate PA (h/w)																		
0	1333	1.0		690	1.0		137	1.0		233	1.0		1141	1.0		614	1.0	
≤20	578	0.93(0.76-1.14)	0.485	317	0.98(0.83-1.17)	0.834	70	1.12(0.82-1.53)	0.483	97	0.95(0.73-1.24)	0.720	451	0.86(0.75-0.99)	0.040	239	0.87(0.73-1.04)	0.120
>20	895	0.78(0.65-0.95)	0.012	541	0.93(0.79-1.09)	0.360	124	1.05(0.79-1.40)	0.737	118	0.69(0.54-0.90)	0.005	743	0.90(0.79-1.03)	0.119	458	0.95(0.81-1.12)	0.560
trend		0.89(0.81-0.98)	0.014		0.97(0.89-1.04)	0.379		1.03(0.89-1.19)	0.693		0.84(0.74-0.96)	0.007		0.94(0.88-1.01)	0.076		0.97(0.90-1.05)	0.449
Light PA (h/w)																		
≤14	1377	1.0		764	1.0		166	1.0		202	1.0		1255	1.0		671	1.0	
≤27	347	1.16(0.91-1.48)	0.227	189	0.97(0.79-1.19)	0.770	54	1.24(0.88-1.74)	0.223	44	0.93(0.66-1.33)	0.706	309	1.01(0.86-1.19)	0.903	175	1.02(0.84-1.25)	0.823
>27	1081	1.21(1.00-1.46)	0.047	596	0.97(0.83-1.14)	0.728	111	0.86(0.65-1.16)	0.324	201	1.26(0.99-1.60)	0.065	770	0.80(0.70-0.91)	0.001	465	0.84(0.72-0.99)	0.037
trend		1.10(1.00-1.21)	0.042		0.99(0.91-1.07)	0.703		0.94(0.82-1.09)	0.431		1.12(0.99-1.26)	0.073		0.90(0.84-0.96)	0.001		0.92(0.85-0.99)	0.047
Total PA (MET-h/w)																		
≤212.2	1106	1.0		647	1.0		138	1.0		199	1.0		821	1.0		518	1.0	
≤342.7	884	0.71(0.58-0.86)	0.001	503	0.86(0.73-0.99)	0.056	104	0.81(0.61-1.07)	0.141	139	0.74(0.57-0.95)	0.016	775	0.90(0.78-1.03)	0.124	427	0.82(0.70-0.97)	0.017
>342.7	817	0.62(0.50-0.76)	<0.001	399	0.68(0.58-0.81)	<0.001	89	0.81(0.59-1.10)	0.174	110	0.61(0.46-0.80)	<0.001	739	0.81(0.70-0.93)	0.003	366	0.69(0.58-0.82)	<0.001
trend		0.79(0.71-0.87)	<0.001		0.83(0.76-0.90)	<0.001		0.89(0.76-1.04)	0.150		0.78(0.68-0.90)	<0.001		0.90(0.83-0.96)	0.003		0.83(0.76-0.91)	<0.001
Sitting time (h/w)																		
≤21	938	1.0		497	1.0		112	1.0		139	1.0		755	1.0		421	1.0	
≤42	1180	0.99(0.83-1.18)	0.917	697	1.23(1.06-1.43)	0.006	133	0.98(0.74-1.29)	0.874	195	1.13(0.89-1.43)	0.317	1005	1.08(0.95-1.22)	0.255	583	1.15(0.99-1.34)	0.066
>42	688	1.27(1.03-1.57)	0.030	355	1.23(1.03-1.47)	0.024	86	1.31(0.96-1.80)	0.089	113	1.06(0.80-1.41)	0.663	574	1.07(0.92-1.24)	0.412	307	1.12(0.93-1.34)	0.236
trend		1.14(1.00-1.24)	0.046		1.12(1.03-1.22)	0.011		1.13(0.97-1.33)	0.128		1.05(0.91-1.20)	0.535		1.04(0.96-1.12)	0.366		1.06(0.97-1.16)	0.177
Watching TV (h/w)																		
≤7	746	1.0		398	1.0		84	1.0		129	1.0		693	1.0		362	1.0	
≤14	1334	1.18(0.99-1.42)	0.071	735	1.05(0.90-1.23)	0.507	160	1.09(0.82-1.45)	0.534	204	0.90(0.71-1.15)	0.410	1122	1.07(0.95-1.22)	0.276	618	1.08(0.92-1.26)	0.352
>14	726	1.44(1.16-1.81)	0.001	416	1.19(0.99-1.43)	0.059	87	1.08(0.78-1.51)	0.645	114	1.04(0.78-1.39)	0.783	519	1.09(0.93-1.27)	0.310	331	1.20(1.00-1.45)	0.052
trend		1.20(1.08-1.34)	0.001		1.09(0.99-1.20)	0.057		1.04(0.88-1.23)	0.651		1.02(0.88-1.18)	0.786		1.05(0.97-1.13)	0.257		1.10(1.00-1.20)	0.053
Sleep duration (h/d)																		
≤7	668	1.0		361	1.0		65	1.0		104	1.0		495	1.0		301	1.0	
≤8	1682	0.99(0.82-1.20)	0.953	946	1.06(0.91-1.25)	0.441	211	1.42(1.05-1.92)	0.024	241	0.89(0.69-1.15)	0.365	1394	1.05(0.92-1.21)	0.459	776	0.99(0.84-1.16)	0.854
>8	457	1.09(0.85-1.40)	0.509	242	1.18(0.95-1.45)	0.135	55	1.57(1.06-2.34)	0.025	103	1.46(1.07-1.99)	0.018	446	1.17(0.98-1.40)	0.078	234	1.11(0.90-1.37)	0.340
trend		1.04(0.92-1.18)	0.523		1.09(0.98-1.21)	0.129		1.26(1.04-1.53)	0.019		1.21(1.02-1.42)	0.027		1.08(0.99-1.18)	0.085		1.05(0.94-1.16)	0.401

All models were adjusted for age at interview, BMI, education, marital status, personal income, occupation, smoking status, drinking status, tea consumption, and intake of red meat, white meat, fish, vegetables, fruits, and soy food, and mutually adjusted for light to vigorous PA categories.

PA: physical activity; MET: metabolic equivalent; HDL-c: high-density lipoprotein cholesterol; SBP: systolic blood pressure; DBP: diastolic blood pressure.

Cases: abnormal MS component (including waist circumference, triglyceride, HDL-c, glucose, SBP and DBP) subjects.

h/w: hours per week; h/d: hours per day; MET-h/w: MET hours per week.

**Table 4:** Associations of MS components with PA and other activities: abnormal MS components vs. normal MS components in women.

	Waist Circumference (cm)			Triglyceride (mmol/L)			HDL-c (mmol/L)			Glucose (mmol/L)			SBP (mmHg)			DBP (mmHg)		
	Cases	OR (95%CI)	p	Cases	OR (95%CI)	p	Cases	OR (95%CI)	p	Cases	OR (95%CI)	p	Cases	OR (95%CI)	p	Cases	OR (95%CI)	p
Vigorous PA (h/w)																		
≤14	2699	1.0		1285	1.0		1526	1.0		360	1.0		1365	1.0		773	1.0	
≤42	2829	0.94(0.82-1.08)	0.401	1319	0.90(0.80-1.01)	0.076	1098	0.82(0.73-0.92)	0.001	350	0.72(0.59-0.87)	0.001	1542	0.82(0.73-0.92)	0.001	756	0.84(0.73-0.97)	0.017
>42	1570	0.83(0.70-0.98)	0.027	709	0.79(0.68-0.91)	0.001	618	0.73(0.63-0.84)	<0.001	172	0.59(0.47-0.76)	<0.001	811	0.79(0.69-0.91)	0.001	391	0.70(0.58-0.83)	<0.001
trend		0.91(0.84-0.99)	0.028		0.90(0.83-0.96)	0.001		0.85(0.80-0.92)	0.001		0.77(0.68-0.87)	<0.001		0.89(0.83-0.95)	0.001		0.84(0.76-0.91)	<0.001
Moderate PA (h/w)																		
0	3457	1.0		1675	1.0		1545	1.0		465	1.0		1822	1.0		934	1.0	
≤14	1562	1.01(0.88-1.15)	0.931	712	0.95(0.85-1.06)	0.374	679	1.04(0.93-1.16)	0.545	191	0.88(0.73-1.06)	0.189	824	0.97(0.87-1.09)	0.650	410	0.94(0.82-1.08)	0.400
>14	2078	0.76(0.67-0.87)	<0.001	926	0.81(0.73-0.91)	<0.001	1018	0.89(0.80-1.00)	0.040	226	0.73(0.60-0.88)	0.001	1072	0.94(0.85-1.05)	0.288	576	0.90(0.79-1.03)	0.118
trend		0.88(0.83-0.94)	<0.001		0.91(0.86-0.96)	<0.001		0.95(0.90-1.00)	0.066		0.86(0.78-0.94)	0.001		0.97(0.92-1.03)	0.286		0.95(0.87-1.01)	0.111
Light PA (h/w)																		
≤14	3313	1.0		1523	1.0		1395	1.0		381	1.0		1771	1.0		887	1.0	
≤28	2086	0.90(0.79-1.01)	0.073	975	0.98(0.88-1.08)	0.628	904	1.03(0.93-1.14)	0.597	279	1.08(0.91-1.29)	0.361	1124	0.89(0.81-0.98)	0.029	559	0.92(0.81-1.05)	0.228
>28	1694	0.92(0.79-1.07)	0.277	815	0.96(0.84-1.08)	0.480	942	1.10(0.97-1.24)	0.131	222	1.00(0.81-1.23)	0.997	836	0.89(0.79-1.01)	0.080	474	0.91(0.78-1.06)	0.235
trend		0.95(0.88-1.02)	0.157		0.98(0.92-1.04)	0.443		1.05(0.98-1.11)	0.149		1.01(0.91-1.12)	0.843		0.94(0.88-0.99)	0.033		0.95(0.88-1.02)	0.172
Total PA (MET-h/w)																		
≤216.2	2324	1.0		1136	1.0		1287	1.0		321	1.0		1197	1.0		670	1.0	
≤334.9	2384	0.86(0.76-0.98)	0.027	1083	0.90(0.80-1.00)	0.054	1017	0.86(0.78-0.96)	0.008	289	0.77(0.64-0.93)	0.007	1244	0.89(0.80-0.99)	0.045	638	0.88(0.76-0.99)	0.058
>334.9	2389	0.81(0.70-0.92)	0.002	1094	0.83(0.74-0.93)	0.002	938	0.75(0.67-0.84)	<0.001	272	0.65(0.54-0.79)	<0.001	1277	0.86(0.77-0.96)	0.010	612	0.75(0.66-0.84)	<0.001
trend		0.90(0.84-0.96)	0.002		0.91(0.86-0.97)	0.002		0.87(0.82-0.92)	<0.001		0.81(0.74-0.89)	<0.001		0.93(0.88-0.98)	0.012		0.87(0.82-0.94)	<0.001
Sitting time (h/w)																		
≤21	2498	1.0		1148	1.0		1225	1.0		270	1.0		1245	1.0		651	1.0	
≤42	2876	1.08(0.96-1.21)	0.187	1336	1.02(0.92-1.12)	0.751	1316	1.02(0.93-1.13)	0.652	356	1.09(0.92-1.29)	0.338	1454	0.93(0.84-1.02)	0.132	785	1.08(0.95-1.22)	0.239
>42	1716	1.37(1.19-1.58)	<0.001	829	1.18(1.05-1.33)	0.007	700	1.13(1.00-1.27)	0.045	255	1.39(1.15-1.69)	0.001	1016	1.13(1.01-1.27)	0.035	482	1.25(1.08-1.45)	0.002
trend		1.16(1.08-1.24)	<0.001		1.08(1.02-1.14)	0.013		1.06(1.00-1.12)	0.065		1.18(1.07-1.30)	0.001		1.05(0.99-1.12)	0.077		1.11(1.04-1.20)	0.003
Watching TV (h/w)																		
≤7	2602	1.0		1168	1.0		1117	1.0		331	1.0		1487	1.0		670	1.0	
≤14	3342	0.92(0.83-1.04)	0.178	1603	1.10(1.00-1.21)	0.066	1555	1.00(0.91-1.10)	0.952	424	1.01(0.87-1.19)	0.861	1702	0.98(0.89-1.08)	0.653	937	1.09(0.97-1.23)	0.156
>14	1150	1.05(0.90-1.23)	0.518	542	1.19(1.04-1.36)	0.011	578	1.06(0.93-1.21)	0.357	127	0.97(0.77-1.22)	0.768	529	1.02(0.89-1.17)	0.778	313	1.15(0.98-1.36)	0.087
trend		1.01(0.93-1.08)	0.892		1.09(1.02-1.16)	0.009		1.02(0.96-1.09)	0.482		0.99(0.89-1.11)	0.877		1.00(0.94-1.07)	0.949		1.08(0.99-1.16)	0.068
Sleep duration (h/d)																		
≤7	1319	1.0		551	1.0		629	1.0		138	1.0		648	1.0		346	1.0	
≤8	4375	1.00(0.88-1.14)	0.983	2077	1.19(1.06-1.34)	0.003	2014	1.04(0.93-1.16)	0.496	532	1.18(0.97-1.45)	0.105	2300	1.03(0.92-1.16)	0.567	1197	1.10(0.96-1.27)	0.184
>8	1403	1.17(0.99-1.38)	0.064	685	1.29(1.12-1.49)	<0.001	599	1.12(0.98-1.29)	0.103	212	1.42(1.12-1.80)	0.003	770	0.99(0.86-1.13)	0.836	377	1.10(0.92-1.31)	0.282
trend		1.08(0.99-1.18)	0.066		1.13(1.06-1.22)	0.001		1.06(0.99-1.13)	0.110		1.20(1.07-1.35)	0.002		0.99(0.93-1.06)	0.826		1.05(0.96-1.14)	0.280

All models were adjusted for age at interview, BMI, education, marital status, personal income, occupation, smoking status, drinking status, tea consumption, and intake of red meat, white meat, fish, vegetables, fruits, and soy food, and mutually adjusted for light to vigorous PA categories.

PA: physical activity; MET: metabolic equivalent; HDL-c: high-density lipoprotein cholesterol; SBP: systolic blood pressure; DBP: diastolic blood pressure.

Cases: abnormal MS component (including waist circumference, triglyceride, HDL-c, glucose, SBP and DBP) subjects.

h/w: hours per week; h/d: hours per day; MET-h/w: MET hours per week.

## Discussion

In our study we found that women had a higher prevalence of MS than men in rural areas. Moderate and vigorous PA each week (or a high MET-h/w) were important protective factors for abnormal MS components and may result in a 10–30% decrease in the odds, while an increase in sitting time per week and sleep hours per day were risk factors for MS components.

In this cross-sectional study among adults in rural China, there was a lower prevalence of MS in men (17.5%) than in women (23.7%). This difference is consistent with a recent large-scale, population-based study in which a significantly higher prevalence of MS was found in Malay women (30.1%) than in men (24.8%) [29]. However, a recent cross-sectional study of 33,149 employees in Northeast China found a higher prevalence of MS in men (24.5%) than in women (15.4%) [30]. The different prevalence between our study and a study in Northeast China might be due to the fact that most of participants in our study were farmers (some females were housewives), and were living in rural areas, while participants in the study of Northeast China were mainly living in urban China with occupations of professionals, clerks and workers. These two populations had different economic status and lifestyles. Also in urban China, the incidence of MS in men reaches its peak during middle-age and beyond, while the incidence of MS in women increases over age of 60 years [31].

Little is known about the association of total PA with MS in rural China. A lower prevalence of MS in the highest tertile of total PA has been reported with ORs of 0.55 for men and 0.63 for women, compared with those in the lowest tertile in Portuguese adults [14]. No such association was found in a study of Costa Rican adults [15]. In our study we found that the highest tertile of total PA was associated with a reduced risk of MS (OR: 0.59 for men and 0.75 for women), compared with those in the lowest tertile of MET. We also found that the highest tertile of hours in vigorous PA was associated with 47% and 30% decreased odds of MS in men and women, respectively. For those in the highest tertile of hours in moderate PA was associated with a 10% decreased odds of MS among women only. These results were consistence with that in Esteghamati’s study in which they concluded that longer durations of moderate and vigorous physical activity are associated with a reduced occurrence of MS [32]. A possible mechanism for this relationship is that the effects of exercise-induced mitochondrial biogenesis in skeletal muscle, which represents about 80–90% of all insulin sensitive tissues and accounts for approximately 50% of basal metabolic rate [33]. The increase in mitochondrial biogenesis (increased volume and functional capacity) is fundamentally important, as it leads to greater rates of oxidative phosphorylation, and an improved capacity to utilize fatty acids during sub-maximal exercise [34]. Additionally, PA also improves insulin sensitivity directly by transporters in both muscle and adipose tissue, which protects against MS.

Previous reports have shown that high levels of PA play a critical role in decreasing the risks of high triglyceride, high glucose, low HDL-c [35–39], and abdominal obesity [40]. However, several studies did not find this relationship [10, 41]. In our study we observed that the highest tertile of total PA (> 334.9 MET-h/w for women and 342.7 MET-h/w for men), as well as vigorous PA (> 42 h/w), was associated with a 15–40% decrease in the odds of all these abnormal MS components in both genders, with an exception of HDL-c in men, compared with those in the lowest tertile. We also found that the highest tertile of moderate PA (> 14 h/w for women and 20 h/w for men) was associated with a 20–30% decrease in the odds of high waist circumference and high glucose in both genders, and with a 19% decrease in the odds of high triglyceride only in women. Our data are in line with several studies. Di Loreto reported that constant energy expenditure > 20 METs/h per week decreased waist circumference and triglyceride and increased HDL-c, which therefore, was associated with a 4–5% decrease in the 10-year coronary heart disease risk [42]. Another study reported that higher levels of total PA were associated with a significant decrease in the prevalence of central obesity, dyslipidemia, and dysglycemia [32]. However, we didn’t find any association between light-intensity activity and waist circumference or blood glucose, which were inconsistent with previous findings [15, 43, 44]. It could be that light-intensity level is too week to improve insulin sensitivity and then it is hard to detect its effect on MS components [45]. In addition, different research populations may be another reason for these inconsistencies. Therefore, intervention trials using objective measures of PA are required to validate our results and reveal the physiological and behavioral mechanisms underlying the observations.

Previous studies have also demonstrated that PA has protective effects against hypertension [35, 37, 39, 46]. While this protective effect was not to be found in rural people in South Africa [47], or in Taiwanese adults regardless of PA intensity [10]. However, we observed that increased hours of vigorous PA was associated with a 20–40% reduced odds of high SBP and DBP, and that increasing the hours spent in light PA may be associated with about 10% decreased odds of high SBP in both genders.

It has been shown that lifestyles have changed over the preceding decades in developing countries, especially in China. There has been a trend towards an increase in sedentary lifestyles that likely contribute to an increased incidence of MS [14]. Also it is important to highlight that many observational evidence between sedentary behavior and MS components is complex, depending on the type of sedentary behavior. An overview of systematic reviews reported that there are strong associations between sedentary behavior (based on watching TV) and obesity and blood pressure; and there are strong associations between sedentary behavior (based on watching TV, sitting time) and MS and type 2 diabetes [48]. So we focus on the association of three particular sedentary behavior (sitting, watching TV and sleep duration) on MS and its components among people in rural China and have drawn divergent conclusions. We found more sitting time was associated with the 16% increased odds of MS in both men and women, which were consistent with a meta-analysis that more sitting time has been shown to increase 73% of MS odds [18], but this association cannot be found in a cohort study among American men [49]. But in current study we further found more sitting time was associated with higher waist circumference in men and higher triglyceride in women, which were consistent with the study in American men [49].

Watching TV, another sedentary behaviors, was positively associated with high triglyceride and high glucose, (ORs of 2.59 and 13.9 respectively for watching TV ≥ 21h/week vs. 0–5 h/week) in Taiwanese adults [10] and associated with central obesity in rural Indian women [50]. We found that watching TV over 14h/w was associated with a risk of high waist circumference in men (OR = 1.20) and high triglyceride in women (OR = 1.09), compared with people watching TV 7 hours per week or less. A potential mechanisms of this association could be that a significant reduction lipoprotein lipase activity in the microvascular beds in muscle, a key enzyme regulating lipid metabolism, has been shown to occur during sedentary activity [51]. Additionally, sedentary behavior, simply on the basis of its low expenditure, may result in being overweight or obese [52]. We found that sleeping for more than 8 hours was associated with a 20.13% attributable risk of low HDL-c, a 16.83% attributable risk of high glucose in men, and a 16.69% attributable risk of high glucose, a 12.02% attributable risk of high triglyceride in women, compare to those sleeping less than 7 hours per day. A previous study in Costa Rica also got similar conclusions [14]. More studies need to be granted for further investigation in association between sleeping and MS components and underline mechanisms.

The strengths of our study include a population-based design, a large sample size, and a comprehensive assessment of associations of a wide range of PA and other lifestyle factors with MS and its components. However, limitations should be taken into account. First, our data on PA and other lifestyles were obtained by self-report, which could bias the results. However, PA and sitting time were assessed with International Physical Activity Questionnaire, a valid and reliable instrument, and may control errors to some extent. Second, the cross-sectional design cannot get any conclusions on the causality of the observed associations. Thus, longitudinal studies should be used in the future to evaluate potentially causal relationships.

## Conclusions

In conclusion, we found there were an independent preventive effect of total PA, moderate and vigorous PA and a risk effect of sitting time on MS and its components. A long sleep duration was also shown to be related to abnormal serum glucose and lipids. Our findings suggest that participation in PA and decrease of sedentary time should be encouraged to reduce the prevalence of the MS and its abnormal components, even in rural China. In the absence of contraindications, more vigorous PA should also be considered to obtain additional health benefits.

## Supporting Information

S1 Table Attributable risk (CI, %) of metabolic syndrome and its components by PA and other activities among rural men. (DOCX)

S2 Table Attributable risk (CI, %) of metabolic syndrome and its components by PA and other activities among rural women. (DOCX)
