# Supplementary material for: Physical Activity and Sedentary Behavior Associated with Components of Metabolic Syndrome among People in Rural China
Source: PLoS One. 2016 Jan 20;11(1):e0147062. doi: 10.1371/journal.pone.0147062 (PMC4720370; doi:10.1371/journal.pone.0147062)
Supplement: S1 Table — (DOCX) [file pone.0147062.s001.docx]

S1 table. Attributable risk (CI, %) of metabolic syndrome and its components by PA and other activities among rural men

|  | Metabolic Syndrome | Waist Circumference | Triglyceride | HDL-c | Glucose | SBP | DBP |
| --- | --- | --- | --- | --- | --- | --- | --- |
| Vigorous PA (h/w) | |  |  |  |  |  |  |
| ≤7 | 0 | 0 | 0 | 0 | 0 | 0 | 0 |
| ≤42 | -21.06(-31.44-(-10.85)) | -21.40(-33.38-(-9.33)) | -15.57(-25.32-(-5.95)) | -7.63(-24.44-8.94) | -17.25(-31.53-(-2.23)) | -11.76(-19.49-(-3.82)) | -14.31(-23.53-(-4.55)) |
| >42 | -27.38(-36.10-(-18.41)) | -24.91(-35.63-(-13.22)) | -21.25(-30.23-(-12.23)) | -12.36(-28.21-4.80) | -16.55(-30.48-(-2.34)) | -16.21(-23.30-(-8.81)) | -23.49(-31.80-(-14.32)) |
| Moderate PA (h/w) | |  |  |  |  |  |  |
| 0 | 0 | 0 | 0 | 0 | 0 | 0 | 0 |
| ≤20 | -2.21(-7.63-3.85) | -2.22(-8.04-4.16) | -0.62(-5.55-5.00) | 3.56(-5.87-14.03) | -1.57(-9.09-6.89) | -4.63(-8.58-(-0.32)) | -4.22(-9.19-1.23) |
| >20 | -7.26(-14.54-0.00) | -10.35(-17.54-(-2.18)) | -3.01(-9.60-3.62) | 2.07(-9.72-14.45) | -15.23(-24.39-(-4.45)) | -4.53(-10.01-1.28) | -2.15(-8.70-4.81) |
| Total PA (MET-h/w) | |  |  |  |  |  |  |
| ≤212.2 | 0 | 0 | 0 | 0 | 0 | 0 | 0 |
| ≤342.7 | -15.00(-23.92-(-5.51)) | -18.75(-29.65-(-8.25)) | -7.99(-16.65-(-0.53)) | -11.05(-25.67-3.54) | -15.74(-29.02-(-2.69)) | -5.34(-12.79-1.52) | -10.43(-18.68-(-1.60)) |
| >342.7 | -28.52(-39.17-(-17.86)) | -27.60 (-39.79-(-15.82)) | -21.54(-30.31-(-11.76)) | -11.49(-28.61-5.15) | -26.87(-41.50-(-12.19)) | -11.38(-19.24-(-3.91)) | -20.44(-29.86-(-10.93)) |
| Sitting time (h/w) | |  |  |  |  |  |  |
| ≤21 | 0 | 0 | 0 | 0 | 0 | 0 | 0 |
| ≤42 | 10.31(1.08-19.41) | -0.56(-10.39-9.06) | 11.10(3.15-18.92) | -1.11(-16.62-13.72) | 6.63(-6.39-19.01) | 4.17(-2.79-10.69) | 7.56(-0.55-15.63) |
| >42 | 11.38(3.64-19.46) | 9.19(1.11-17.61) | 8.06(1.13-15.19) | 10.45(-1.53-23.14) | 2.21(-8.15-13.39) | 2.53(-3.06-8.18) | 4.34(-2.71-11.39) |
| Watching TV (h/w) | |  |  |  |  |  |  |
| ≤7 | 0 | 0 | 0 | 0 | 0 | 0 | 0 |
| ≤14 | -1.83(-12.10-8.75) | 9.47(-0.58-19.62) | 2.88(-6.32-12.03) | 5.10(-12.06-21.20) | -6.38(-21.04-8.25) | 4.00(-3.07-11.59) | 4.55(-5.01-13.41) |
| >14 | 6.24(-1.59-14.68) | 13.52(5.38-22.35) | 6.70(-0.38-13.99) | 3.02(-9.37-16.58) | 1.55(-9.45-13.27) | 3.43(-2.84-9.64) | 7.17(0.00-14.81) |
| Sleep duration (h/d) | |  |  |  |  |  |  |
| ≤7 | 0 | 0 | 0 | 0 | 0 | 0 | 0 |
| ≤8 | -2.26(-16.35-10.58) | -0.75(-15.50-12.97) | 4.25(-7.15-15.64) | 23.65(3.56-40.43) | -8.86(-29.76-9.99) | 3.53(-6.23-13.34) | -0.74(-13.41-10.57) |
| >8 | 8.13(-2.26-18.71) | 4.01(-7.49-15.67) | 7.53(-2.31-16.91) | 20.13(2.59-37.21) | 16.83(2.99-30.33) | 6.74(-0.86-14.54) | 4.63(-4.62-14.05) |

PA: physical activity; MET: metabolic equivalent; HDL-c: high-density lipoprotein cholesterol; SBP: systolic blood pressure; DBP: diastolic blood pressure.
Minus sign (-): the decrease of attributable risk (exposed group vs. control group).
h/w: hours per week; h/d: hours per day; MET-h/w: MET hours per week.
