# Supplementary material for: Physical Activity and Sedentary Behavior Associated with Components of Metabolic Syndrome among People in Rural China
Source: PLoS One. 2016 Jan 20;11(1):e0147062. doi: 10.1371/journal.pone.0147062 (PMC4720370; doi:10.1371/journal.pone.0147062)
Supplement: S2 Table — (DOCX) [file pone.0147062.s002.docx]

S2 table. Attributable risk (CI, %) of metabolic syndrome and its components by PA and other activities among rural women

|  | Metabolic syndrome | Waist Circumference | Triglyceride | HDL-c | Glucose | SBP | DBP |
| --- | --- | --- | --- | --- | --- | --- | --- |
| Vigorous PA (h/w) | |  |  |  |  |  |  |
| ≤14 | 0 | 0 | 0 | 0 | 0 | 0 | 0 |
| ≤42 | -7.93(-13.57-(-2.35)) | -2.61(-8.27-3.28) | -4.78(-10.03-0.45) | -9.46(-14.89-(-3.99)) | -14.72(-23.13-(-6.33)) | -8.66(-13.58-(-3.67)) | -7.87(-14.03-(-1.39)) |
| >42 | -11.46(-15.88-(-6.96)) | -5.66(-10.45-(-0.63)) | -7.67(-12.18-(-3.15)) | -10.79(-15.40-(-6.12)) | -16.34(-22.18-(-8.96)) | -7.47(-11.43-(-3.07)) | -11.46(-16.81-(-6.19)) |
| Moderate PA (h/w) | |  |  |  |  |  |  |
| 0 | 0 | 0 | 0 | 0 | 0 | 0 | 0 |
| ≤14 | -1.23(-4.77-2.37) | 0.30(-3.70-4.27) | -1.53(-4.75-1.78) | 1.19(-2.16-4.61) | -3.79(-8.95-1.79) | -0.92(-4.09-2.65) | -1.86(-5.78-2.37) |
| >14 | -8.93(-13.49-(-4.27)) | -11.26(-16.16-(-5.80)) | -8.51(-12.54-(-3.86)) | -4.70(-8.88-0.00) | -12.46(-19.65-(-5.18)) | -2.54(-6.61-2.02) | -4.25(-9.37-1.21) |
| Total PA (MET-h/w) | |  |  |  |  |  |  |
| ≤216.2 | 0 | 0 | 0 | 0 | 0 | 0 | 0 |
| ≤334.9 | -5.42(-10.89-(-0.47)) | -6.53(-11.75-(-0.88)) | -4.88(-10.27-0.00) | -7.25(-11.89-(-1.97)) | -12.03(-20.20-(-3.38)) | -5.31(-10.09-(-0.46)) | -5.92(-12.58-(-0.47)) |
| >334.9 | -13.79(-19.73-(-8.41)) | -9.46(-15.80-(-3.78)) | -8.91(-14.29-(-3.48)) | -14.42(-19.95-(-8.77)) | -20.41(-28.67-(-11.32)) | -7.04(-12.12-(-1.92)) | -13.74(-19.67-(-8.38)) |
| Sitting time (h/w) | |  |  |  |  |  |  |
| ≤21 | 0 | 0 | 0 | 0 | 0 | 0 | 0 |
| ≤42 | 3.43(-2.07-8.79) | 3.86(-2.05-9.55) | 1.01(-4.23-5.74) | 1.02(-3.72-6.25) | 4.37(-4.23-12.84) | -3.67(-8.82-1.00) | 3.90(-2.60-10.03) |
| >42 | 10.57(6.32-15.18) | 10.71(5.80-15.83) | 5.75(1.67-10.07) | 4.31(0.00-8.55) | 11.61(4.81-18.85) | 4.05(0.32-8.06) | 7.74(2.61-13.12) |
| Watching TV (h/w) | |  |  |  |  |  |  |
| ≤7 | 0 | 0 | 0 | 0 | 0 | 0 | 0 |
| ≤14 | -1.16(-6.71-4.37) | -4.79(-10.76-2.23) | 5.37(0.00-10.64) | 0.00(-5.37-5.36) | 0.53(-7.49-9.24) | -1.17(-6.81-4.43) | 4.89(-1.74-11.61) |
| >14 | 1.54(-2.57-14.24) | 1.54(-3.24-6.73) | 5.55(1.22-10.01) | 1.78(-2.15-5.95) | -0.95(-7.78-6.46) | 0.65(-3.73-5.27) | 4.49(-0.63-10.14) |
| Sleep duration (h/d) | |  |  |  |  |  |  |
| ≤7 | 0 | 0 | 0 | 0 | 0 | 0 | 0 |
| ≤8 | 5.04(-4.76-13.73) | 0.00(-9.97-9.56) | 12.54(4.33-20.41) | 2.95(-5.61-10.83) | 11.99(-2.32-25.41) | 2.22(-6.44-10.79) | 7.03(-3.12-16.96) |
| >8 | 7.11(0.00-14.35) | 7.38(-0.47-15.12) | 12.02(5.35-18.76) | 5.47(-0.97-12.27) | 16.69(5.42-27.63） | -0.47(-7.07-5.78) | 4.54(-3.96-12.86) |

PA: physical activity; MET: metabolic equivalent; HDL-c: high-density lipoprotein cholesterol; SBP: systolic blood pressure; DBP: diastolic blood pressure.
Minus sign (-): the decrease of attributable risk (exposed group vs. control group).
h/w: hours per week; h/d: hours per day; MET-h/w: MET hours per week.
